# Concealed Malignancy as a Rare Cause of Refractory Lactic Acidosis: A Fatal Case

**DOI:** 10.7759/cureus.18602

**Published:** 2021-10-08

**Authors:** Mahammed Z Khan Suheb, Wasey Ali Yadullahi Mir, Farheen Naaz, Dhan B Shrestha, Anuj K Paudel

**Affiliations:** 1 Department of Internal Medicine, University of Nebraska Medical Center, Omaha, USA; 2 Department of Internal Medicine, Mount Sinai Hospital, Chicago, USA; 3 Department Internal Medicine, Deccan College of Medical Sciences, Hyderabad, IND; 4 Department of Emergency Medicine, Metrocity Hospital and Research Center, Pokhara, NPL

**Keywords:** lactic acidosis, shock, hypotension, hypoxia, neoplasms

## Abstract

Lactic acidosis is a state in which there is a buildup of lactate in the body to form an excessively low pH in the blood. Elevated lactate levels are often thought to be indicative of relative tissue hypoxia or type A lactic acidosis. Shock, severe anemia, and thromboembolic events can all cause elevated lactate due to tissue hypoperfusion. Malignancy can also lead to an elevation in lactate, a phenomenon described as type B lactic acidosis. Here, we report a case of a 66-year-old male with elevated lactate level, which was refractory to medical treatment. Despite adequate management, including continuous renal replacement therapy, the lactate continued to rise, and consequently, the patient died due to cardiac arrest. Type B lactic acidosis must be considered in patients with elevated lactate levels without hypotension as it has a high mortality rate.

## Introduction

Lactic acidosis is the most common cause of raised anion gap acidosis in intensive care units. Lactic acidosis is defined as a serum lactate level of more than 4 mmol/L and serum pH<7.35. Lactate accumulates due to an increase in pyruvate production, reduced pyruvate conversion to carbon dioxide and water or glucose, and an altered redox state within the cell, which shifts the pyruvate/lactate ratio towards lactate. Based on pathophysiology, there are two types of lactic acidosis [1]. Type A lactic acidosis is due to marked tissue hypoperfusion resulting from hypovolemia, cardiac failure, sepsis, or cardiac arrest [2]. However, in type B lactic acidosis, there is no evidence of systemic hypoperfusion. The mechanisms of type B are toxin-induced impairment of cellular metabolism and regional areas of ischemia. Malignancy, diabetes mellitus, drugs, alcoholism, and mitochondrial dysfunction cause type B lactic acidosis. Here, we present a case of type B lactic acidosis secondary to intravascular large B cell lymphoma.

## Case presentation

A 66-year-old male presented to the medical center with a 12-week history of fatigue, intermittent fevers, pedal edema, and a two-week history of dysuria, urgency, and hesitancy. Physical examination revealed splenomegaly. Other than splenomegaly, other examination findings including vital signs were essentially normal. There was no rash or swelling of the joints. Initial laboratory findings revealed hemoglobin of 10.4 g/dL, platelets 12,9000/uL, and peripheral blood smear showed microcytic anemia without schistocytes. Renal function test showed elevated serum creatinine (2.4 mg/dL) and urea (65 mg/dL). Urinalysis showed hematuria and proteinuria (500 mg/dL). Liver function test (LFT) showed elevated liver enzymes.

**Figure 1:**
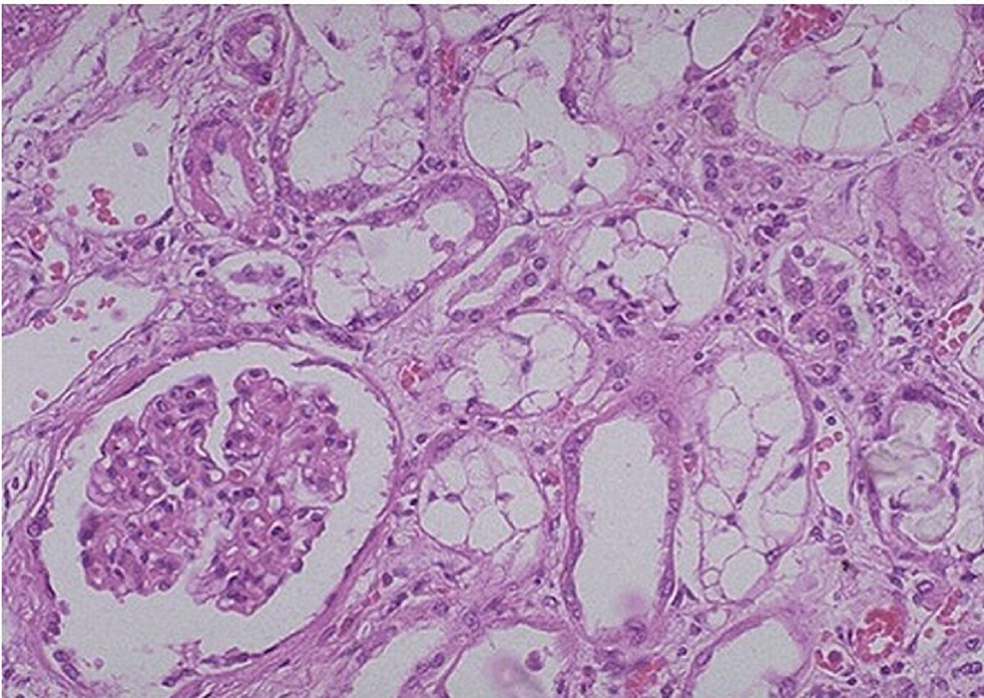
Renal biopsy histopathological image showing acute tubular necrosis

There was a progressive worsening change to different organ systems, including liver, renal, hematopoietic, and inflammatory markers (Table 1). Due to concern of myeloma with deteriorating renal function, the serum electrophoresis was done and it was unremarkable. However, the renal biopsy showed features suggestive of acute tubular necrosis (ATN) (Figure 1). The culture and infectious etiology, along with the autoimmune workup, were negative. The bone marrow biopsy was negative for malignancy and other disorders.

**Table 1:** Laboratory findings showing the progressive worsening of different organ systems including liver, renal, hematopoietic system, and inflammatory markers BUN: blood urea nitrogen, ALT: alanine aminotransferase, AST: aspartate aminotransferase, SGOT: serum glutamic oxaloacetic transaminase, SGPT: serum glutamic pyruvic transaminase, LDH: lactate dehydrogenase, INR: international normalized ratio, L: low, H: high

	Day 1	Day 3	Day 5	Day 7	Day 8	Day 9	Day 10
Bicarbonate (mEq/L)	18 (L)	16 (L)	17 (L)	8 (L)	17 (L)	11 (L)	10 (L)
Anion Gap	11	14	13	24 (H)	21 (H)	30 (H)	36 (H)
BUN (mg/dL)	36 (H)	67 (H)	72 (H)	89 (H)	66 (H)	45 (H)	26 (H)
Creatinine (mg/dL)	1.60 (H)	2.53 (H)	2.8	3.19 (H)	2.17 (H)	1.62	1.13
Bilirubin Total (mg/dL)	0.8	1.6 (L)	1.4 (H)	1.7 (H)		2.6 (H)	4.4 (H)
Alkaline Phosphatase (IU/L)	338 (H)		368 (H)	277		232 (H)	571 (H)
ALT-SGPT (U/L)	55	31 (L)	50	28		41	3397 (H)
AST-SGOT (U/L)	58 (H)	26 (L)	66 (H)	25		67 (H)	11356 (H)
LDH Total (U/L)						1572	
INR			1.9 (L)		2.4 (H)	8.0 (H)	7.1 (H)
Ferritin (ug/mL)		6384 (H)		5122 (H)			8205 (H)

**Figure 2:**
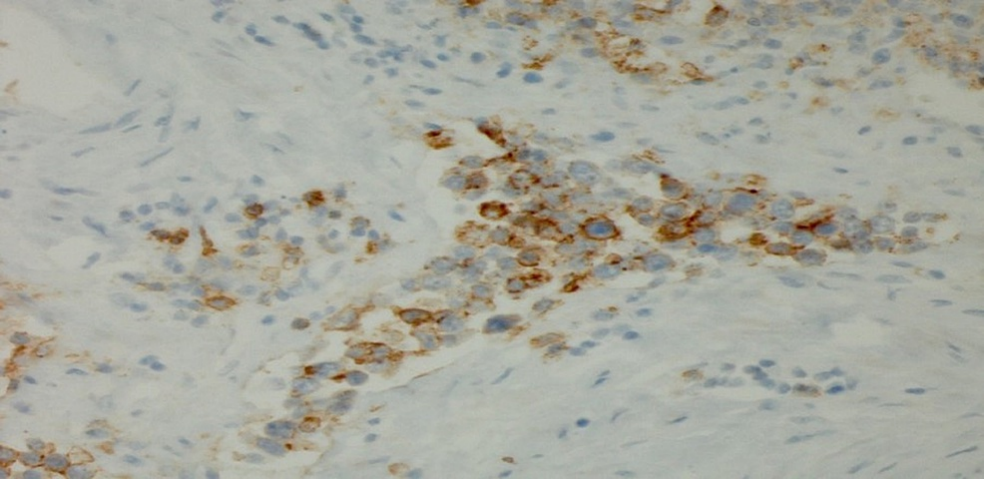
Kidney-CD20 immunostain showing an activated glycosylated phosphoprotein expressed on the surface of all B cells CD: cluster of differentiation

**Figure 3:**
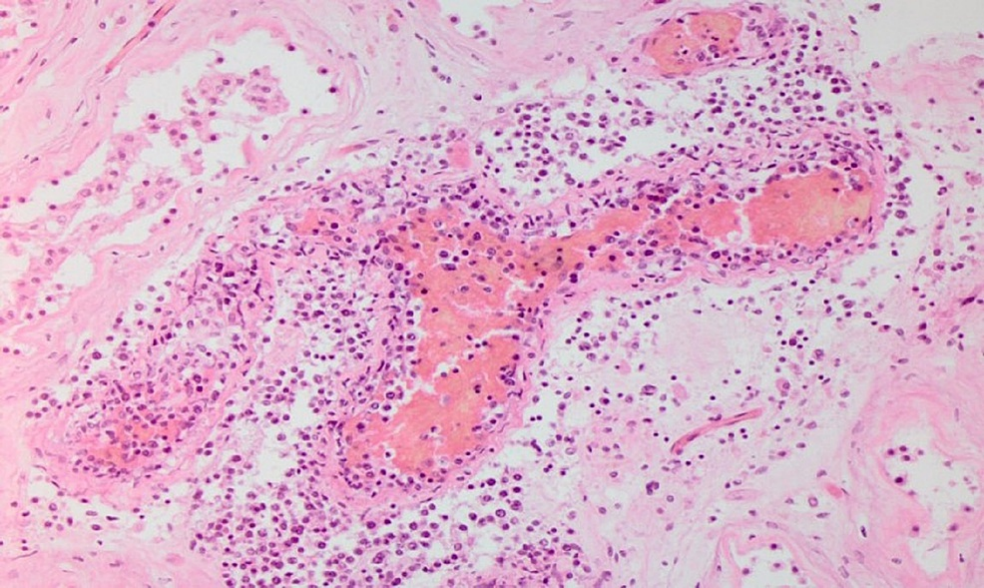
Hematoxylin and eosin stain depicting the malignant cells within a small blood vessel and vessel wall

**Figure 4:**
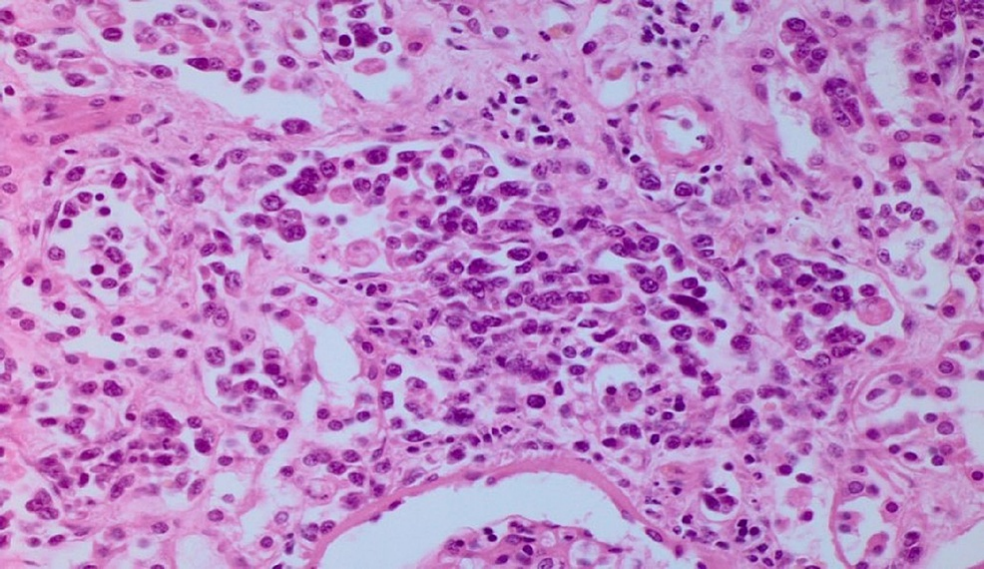
Hematoxylin and eosin stain depicting the malignant lymphoid cells in the vasculature of the kidney on autopsy

The patient was managed with intravenous fluids, antibiotics, and a course of steroids due to concerns for secondary hemophagocytic lymphohistiocytosis (HLH). However, the lactic acid level continued to rise. In search for the potential source of localized organ ischemia, CT imaging of abdomen and pelvis with angiography of the mesenteric vessels was performed, but it was negative. On day seven, the patient deteriorated with hypoxic respiratory failure and shock, needing mechanical ventilation with vasopressor support. The patient had declining kidney function, metabolic acidosis, and anasarca; continuous renal replacement (CRRT) was initiated. Despite CRRT, lactate continued to rise, peaking at 26 mmol/L. His shock state persisted despite several interventions. Consequently, the patient experienced a fatal cardiac arrest. The autopsy revealed intravascular large B cell lymphoma (IVLBCL) in the vasculature of the kidney (Figures 1-4).

## Discussion

Intravascular large B cell lymphoma is a rare subtype of non-Hodgkin lymphoma characterized by the proliferation of lymphoma cells within the lumina of small blood vessels, particularly capillaries and post-capillaries [3]. The clinical presentation varies and often includes symptoms related to organ dysfunction caused by occlusion of the blood vessels. These include constitutional B symptoms (i.e., fever, night sweats, and weight loss), rapidly progressive neurological signs (e.g., dementia, progressive cerebral vascular accidents, peripheral neuropathy), and skin lesions. Bone marrow biopsies can be expected to be normal in IVLBCL, and random skin biopsies often help reach the diagnosis [4].

Type B lactic acidosis is a rare paraneoplastic phenomenon associated with hematologic and solid malignancies and has a poor prognosis if not treated on time [5]. The causes of type B lactic acidosis from malignancy are multifactorial, including altered lactate metabolism from liver and kidney dysfunction and lactate production by tumor cells due to growth factors promoting a high rate of glycolysis despite adequate tissue oxygenation (Warburg effect) [6]. This occurs because the malignant cells adapt to low-oxygen environments within tumors or because of the relative efficiency of glycolysis of tumor cells. It provides most of the metabolic products for cells to proliferate. With increased end-product pyruvate, there is also increased lactate, which leads to type B lactic acidosis [7]. Persistently, high lactate level despite treatment in type B lactic acidosis is associated with decreased survival in patients with malignancy [6].

This case report illustrates that type B lactic acidosis must be considered in situations of elevated lactate without hypotension during the initial admission. Fever, hyperferritinemia, elevated C-reactive protein (CRP) and serum lactate dehydrogenase (LDH), and kidney failure initially pointed towards an infectious etiology. Cultures and imaging findings ruled out infection and ischemia. However, his lactic acidosis continued to worsen with a clinical course complicated by hypoxic failure and circulatory shock. Despite attempts at resuscitation, lactate remained persistently elevated. Even with adequate management, including continuous renal replacement therapy, the lactate continued to rise, and consequently, the patient died due to cardiac arrest.

## Conclusions

Type B lactic acidosis must be considered when there is an increase in lactate without hypotension. Elevated lactate has been associated with increased mortality in patients with hematological malignancies. Clinicians should consider hematologic malignancies and secondary hemophagocytic lymphohistiocytosis (HLH) in patients with refractory lactic acidosis and high ferritin since rapid clinical deterioration can be fatal.
